# New Diethyl Ammonium Salt of Thiobarbituric Acid Derivative: Synthesis, Molecular Structure Investigations and Docking Studies

**DOI:** 10.3390/molecules201119710

**Published:** 2015-11-19

**Authors:** Assem Barakat, Abdullah Mohammed Al-Majid, Saied M. Soliman, Gehad Lotfy, Hazem A. Ghabbour, Hoong-Kun Fun, Abdul Wadood, Ismail Warad, Joseph C. Sloop

**Affiliations:** 1 Department of Chemistry, College of Science, King Saud University, P. O. Box 2455, Riyadh 11451, Saudi Arabia; amajid@ksu.edu.sa; 2 Department of Chemistry, College of Science & Arts, King Abdulaziz University, P. O. Box 344,Rabigh 21911, Saudi Arabia; saied1soliman@yahoo.com; 3 Department of Chemistry, Faculty of Science, Alexandria University, P. O. Box 426, Ibrahimia, Alexandria 21321, Egypt; 4 Pharmaceutical Organic Chemistry Department, Faculty of Pharmacy, Suez Canal University, Ismailia 41522, Egypt; lotfygehad@yahoo.com; 5 Department of Pharmaceutical Chemistry, College of Pharmacy, King Saud University, P. O. Box 2457, Riyadh 11451, Saudi Arabia; ghabbourh@yahoo.com (H.A.G.); hfun.c@ksu.edu.sa (H.-K.F.); 6 X-ray Crystallography Unit, School of Physics, Universiti Sains Malaysia, Penang 11800, Malaysia; 7 Department of Biochemistry, Abdul Wali Khan University, Mardan-23200, Pakistan; awadood@awkum.edu.pk; 8 Department of Chemistry, Science College, Al-Najah National University, P. O. Box 7, Nablus 0097, Palestine; warad@najah.edu; 9 School of Science and Technology, Georgia Gwinnett College, 1000 University Center Lane, Lawrenceville, GA 30043, USA; jsloop@ggc.edu

**Keywords:** fluorine compound, barbituric acid, DFT, molecular docking simulations

## Abstract

The synthesis of the new diethyl ammonium salt of diethylammonium(*E*)-5-(1,5-*bis*(4-fluorophenyl)-3-oxopent-4-en-1-yl)-1,3-diethyl-4,6-dioxo-2-thioxohexaydropyrimidin-5-ide **3** via a regioselective Michael addition of *N*,*N*-diethylthiobarbituric acid 1 to dienone **2** is described. In **3**, the carboanion of the thiobarbituric moiety is stabilized by the strong intramolecular electron delocalization with the adjacent carbonyl groups and so the reaction proceeds without any cyclization. The molecular structure investigations of **3** were determined by single-crystal X-ray diffraction as well as DFT computations. The theoretically calculated (DFT/B3LYP) geometry agrees well with the crystallographic data. The effect of fluorine replacement by chlorine atoms on the molecular structure aspects were investigated using DFT methods. Calculated electronic spectra showed a bathochromic shift of the π-π* transition when fluorine is replaced by chlorine. Charge decomposition analyses were performed to study possible interaction between the different fragments in the studied systems. Molecular docking simulations examining the inhibitory nature of the compound show an anti-diabetic activity with Pa (probability of activity) value of 0.229.

## 1. Introduction

Barbituric acid derivatives have been studied extensively for their diverse biological activity as antiepileptic, antiviral, antibacterial, antifungal and anti-inflammatory agents. For example, mephobarbital and phenobarbital agents, which incorporate the barbituric acid scaffold, are well known therapies for epilepsy [1,2]. While these therapies are very effective in controlling seizures, major side effects such as hypnosis and sedation often result. Addition of substituted aryl/heterocyclic moieties at the 5-position of the pyrimidinetrione [3,4] or thiopyrimidinetrione [5,6] nucleus dramatically improve the antiepileptic potential.

Because barbituric acid derivatives hold considerable promise as antiviral agents, antitumor agents, sedatives, anti-sclerotics, hypnotics, bacteriostatics, anti-inflammatory agents [7,8], and as urease inhibitors [9], research has been prolific with respect to the preparation and bioactivity studies of numerous barbituric acid analogues [9]. The bioactivities displayed by barbiturates include effecting changes in mental activity ranging from mild sedation and sleep to deep coma. Thus, this class of molecules finds use as anesthetics, as well as in the treatment of migraine headaches, insomnia, anxiety, and seizures [10]. More recently, substituted barbituric acids were synthesized and found to possess analeptic, immunomodulating, anti-cancer, and anti-AIDS activity [11]. Some of these compounds also exhibited selective matrix metalloproteinase (MMP) inhibition [12].

In the last decade, there has been a growing interest and several excellent reviews have reported the value of fluorine incorporation in a variety of pharmaceuticals [13,14,15,16]. It is well known that fluorine and fluorine-containing substituents can significantly alter the properties of organic compounds. The fluorine atom’s size, electronegativity, and electrostatic interactions can often conspire to effect substantial changes in electron density distribution, lipophilicity and omniphobicity at the molecular level [13]. In many instances, the incorporation of fluorine into the molecular architecture results in greater lipophilicity than in other halogenated analogues, leading to an improvement in the compounds’ biological availability. Likewise, it has been shown that pharmacokinetic properties, such as occupation of key positions [14] and oral absorption [15] can be enhanced by the introduction of fluorine atoms into drug structures. This, in turn, can lead to better drug efficacy in blocking metabolic routes of oxidation [16].

It has also been proposed that the nonbonding behavior of covalently bound fluorine may play a critical role in approaching any kind of molecular modeling problem. Thus, as potential fluorinated drugs are designed based on the pharmacore principle, one must account for these interactions when considering the drug’s intended mode of action.

This work addresses the design, regioselective synthesis and characterization by spectroscopic and X-ray diffraction methods of a new fluorinated barbituric acid derivative which incorporates several different pharmacophores in a molecule which may show enhanced biological availability [17,18,19,20,21,22]. Additionally, we seek to provide new insights into the computational modeling of this class of molecule including a substituent effect examination on the molecular structure and properties, absorption spectral simulations to understand the electronic properties of the studied compounds as well as a molecular docking simulation to ascertain potential inhibitory activity of the title compound.

## 2. Results

### Synthesis

**Scheme 1:**
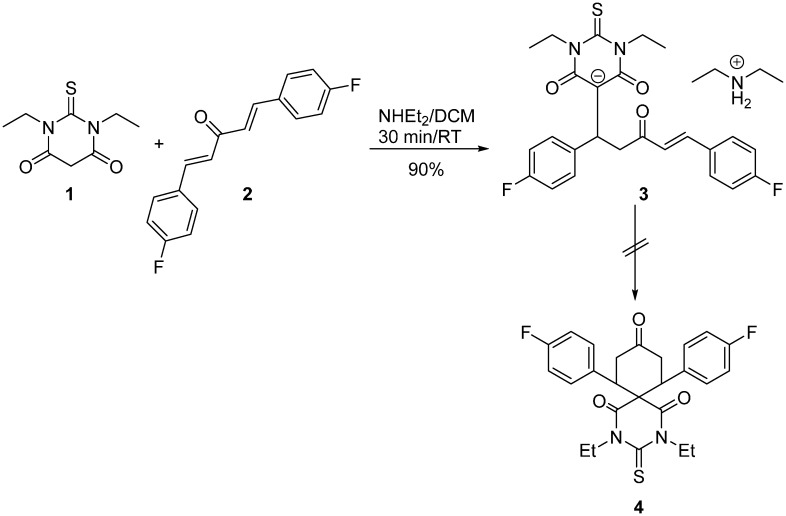
Michael addition reaction of *N*,*N*-diethyl thiobarbituric acid **1** and dienone **2**.

The synthetic pathway for the preparation of **3** is depicted in Scheme 1. Compound **1**, *N*,*N*-diethylthiobarbituric acid, is commercially available, while (*E*)-(1*E*,4*E*)-1,5-*bis*(4-fluorophenyl)penta-1,4-dien-3-one **2** was prepared according to the method described by Islam *et al.* [23]. The reaction of equimolar ratios of **1** with dienone **2** in DCM in the presence of NHEt_2_ as a base gave the desired compound in good overall yield in only 30 min at room temperature. The reaction was highly regiospecific; nucleophilic addition to the carbonyl was not observed. Although the soft nucleophilic nature of the enolate of **1** and the soft electrophilic-carbon of **2**, the Michael-fashion addition gave the ion pair as sole product **3**. It seems that the diethylamine as strong base eliminated the acidic proton of the thiobarbituric ring leading to **3** without any cyclization. The structure of **3** is stabilized by the strong electron delocalization of the negative charge surrounded by the two electron withdrawing carbonyl groups. The compound structure was elucidated by a combined application of ^1^H-NMR, ^13^C-NMR, GC-Mass Spectrometry and X-ray Crystallography.

## 3. Discussion

### 3.1. Single–Crystal X-ray Diffraction Study

The structure of **3** was confirmed by X-ray crystal structural analysis. The crystallographic data, conditions retained for the intensity data collection and some features of the structure refinements are listed in Table 1. The interatomic distances and bond angles are given in Table 2.

**Table 1:** Crystal data, data collection and refinement.

Crystal Data	
Chemical formula	(C_25_H_23_F_2_N_2_O_3_S)·(C_4_H_12_N)·CH_3_OH
Mr	375.12
Crystal system, space group	Triclinic, *P-1*
Temperature (K)	150
*a*, *b*, *c* (Å)	8.9182(4), 9.2271(5), 18.2932(9)
α, β, γ (°)	81.772(2), 80.176(2), 80.538(2)
*V* (Å^3^)	1452.79(13)
*Z*	2
Radiation type	Mo Kα
µ (mm^−1^)	0.16
Crystal size (mm)	0.31 × 0.26 × 0.20
**Data Collection**	
Diffractometer	D8 Venture area detector
Absorption correction	multi-scanSADABS V2014/3
No. of measured, independent and observed [I > 2σ(I)] reflections	72,300, 7527, 5725
*R*_int_	0.055
**Refinement**	
R[*F*^2^ > 2σ(*F*^2^)], *wR*(*F*^2^), *S*	0.050, 0.149, 1.05
No. of reflections	7527
No. of parameters	371
No. of restraints	0
H-atom treatment	H atoms treated by a mixture of independent and constrained refinement
Δρ_max_, Δρ_min_ (e·Å^−3^)	0.66, −0.54

**Table 2:** Selected geometric parameters (Å, °) **3**.

S1–C20	1.6829(17)	N1–C20	1.366(2)
F1–C3	1.356(2)	N1–C22	1.474(2)
F2–C15	1.370(2)	N2–C20	1.369(2)
O1–C9	1.216(2)	N2–C21	1.415(2)
O2–C19	1.254(2)	N2–C24	1.475(2)
O3–C21	1.256(2)	N3–C28	1.488(2)
N1–C19	1.421(2)	N3–C27	1.487(2)
C19–N1–C20	124.08(13)	N1–C19–C18	117.36(14)
C19–N1–C22	116.13(12)	O2–C19–N1	116.20(14)
C20–N1–C22	119.57(13)	O2–C19–C18	126.42(15)
C20–N2–C21	123.59(13)	S1–C20–N2	121.62(12)
C20–N2–C24	119.25(13)	N1–C20–N2	116.11(14)
C21–N2–C24	117.04(13)	S1–C20–N1	122.26(12)
C27–N3–C28	113.94(13)	N2–C21–C18	117.87(14)
F1–C3–C4	118.14(17)	O3–C21–N2	117.81(14)
F1–C3–C2	118.57(17)	O3–C21–C18	124.32(15)
O1–C9–C10	122.01(17)	N1–C22–C23	111.24(14)
O1–C9–C8	122.96(17)	N2–C24–C25	112.24(14)
F2–C15–C14	118.54(17)	N3–C27–C26	110.43(15)
F2–C15–C16	118.58(16)	N3–C28–C29	110.50(15)

**Figure 1:**
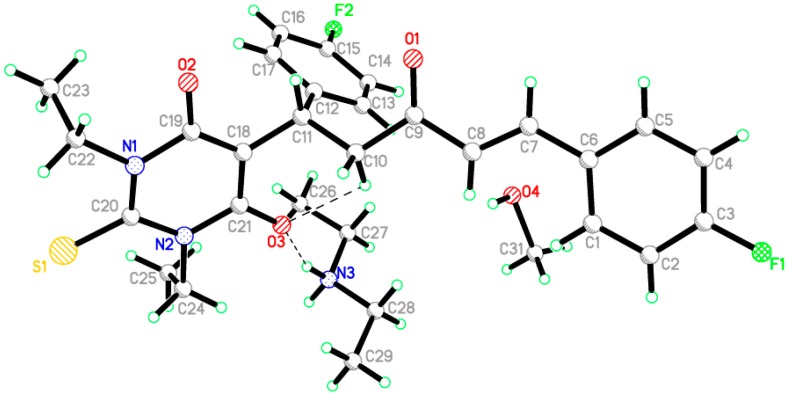
The molecular structure of compound **3** showing 50% probability displacement ellipsoids for non-H atoms, Dotted lines indicate hydrogen bond interactions.

**Figure 2:**
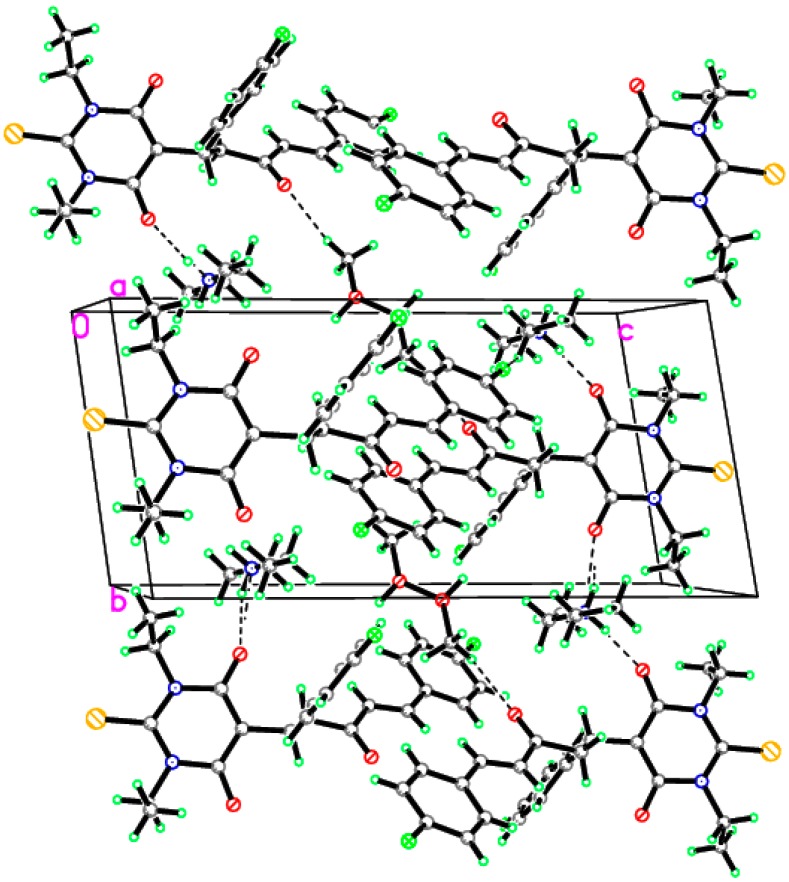
H-bonding network in the crystal structure of **3** viewed along the *b* and *c* axes, Dotted lines indicate intermolecular interactions.

In the unit cell, Figure 1, the titled compound was crystallized with one molecule of diethylamine and one molecule of methanol as solvents and the O3 in the thioxodihydropyrimidine ring form strong hydrogen bond with hydrogen atom of diethyl amine. The thioxodihydropyrimidine-4,6(1*H*,5*H*)-dione ring (C18/C19/N1/C20/N2/C21) was found to adopt an almost mutually perpendicular conformation with one of the phenyl rings (C12-C17) as indicated by a dihedral angle of 87.81(10)°. The thioxodihydropyrimidine ring forms a dihedral angle of 40.87(10)° with the other phenyl ring (C1–C6). The title compound exists in *trans* configuration with respect to the C7=C8 bond [1.334(3) Å]. In the crystal structure, Figure 2, molecules are linked via a network of intermolecular N3–H1N3···O2 and C1–H1A···F2 hydrogen bonds in both axes *b* and *c* (Table 3).

**Table 3:** Hydrogen-bond geometry (Å, °) of **3**.

D–H···A	D–H	H···A	D···A	D–H···A
N3–H1N3···O2 ^i^	0.89(2)	1.83(2)	2.7123(19)	174.3(19)
N3–H3O···O3	0.84(3)	1.89(3)	2.6919(19)	159(2)
C1–H1A···F2 ^ii^	0.9300	2.5100	3.428(2)	171.00
C10–H10B···O3	0.9700	2.4900	3.060(2)	117.00
Symmetry codes: (i) x, y − 1, z; (ii) x − 1, y, z.				

### 3.2. Molecular Modeling Study

**Figure 3:**
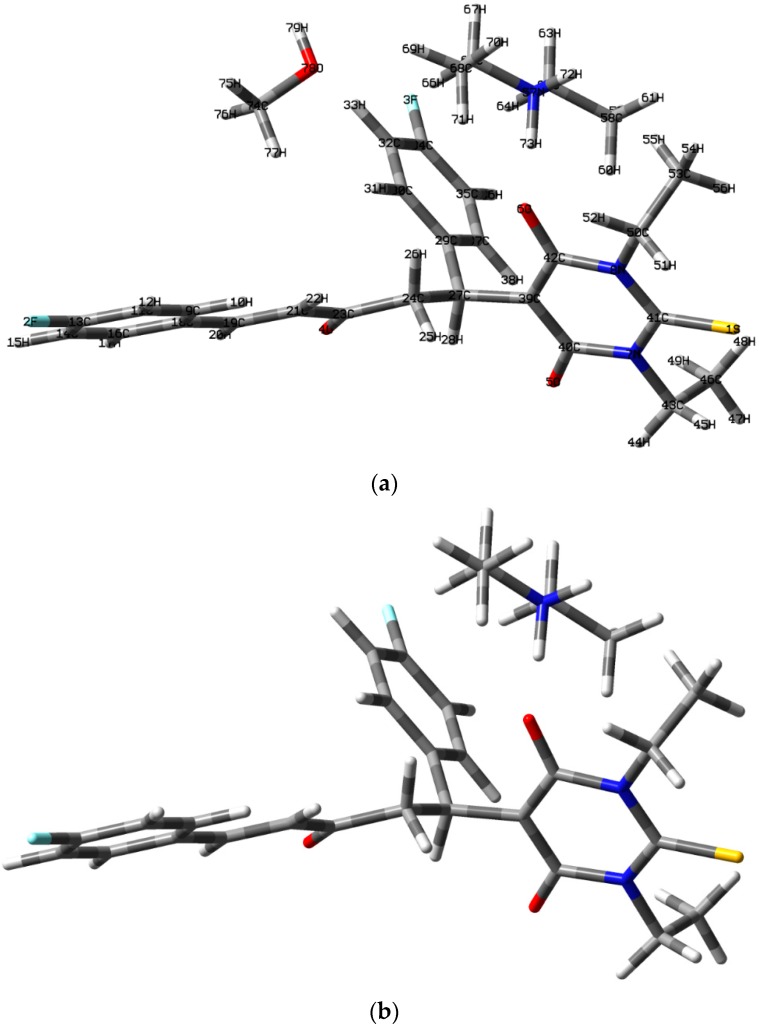

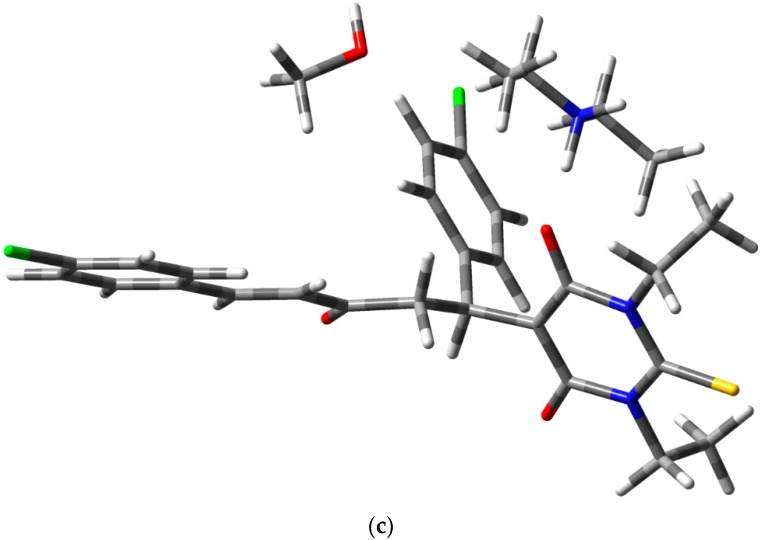
The calculated molecular structure of compounds **3a**–**c**.

In order to predict whether the presence of methanol molecule as well as the type of halogen substituent could affect the molecular and electronic aspects of the studied molecule, we performed DFT computations on the synthesized compound; **3a**, without methanol; **3b** and with Cl-substituent instead of the fluorine one, **3c**. The calculated structures of the studied compounds were shown in Figure 3. The calculated geometrical parameters are listed in Table 4. In comparison with the X-ray structure data of **3a**; the deviations not exceed 0.039 Å (C39–C40) and 2.6° (C27–C39–C42) for the bond length and bond angle values; respectively. The root mean square deviation (RMSD) and the correlation coefficient (*R*^2^) were used to check the accuracy of the DFT results. The RMSD and *R*^2^ values are found to be 0.015 Å, 0.9924 and 1.1°, 0.9619 for the bond lengths and bond angles; respectively. The calculated and experimental molecular structures are in good agreement with each other. Modeling the structure without the methanol molecule (**3b**) showed almost no changes in the geometric parameters (bond distances and bond angles). The methanol has very weak little interaction with the ion pair **3b**. The variations of the bond distance and bond angle values do not exceed 0.003 Å and 0.4°; respectively. These changes in the geometric parameters are insignificant. Furthermore; modeling the structure of the chloro derivative (**3c**) of the studied compound showed no significant change in the geometric parameters.

**Table 4:** The calculated and experimental structural data of the studied compounds using B3LYP/6-31G(d) method.

Parameter ^a^	Calc.			Exp	Parameter ^a^	Calc.			Exp
	a	b	c			a	b	c	
R(1-41)	1.697	1.696	1.696	1.683	A(4-23-24)	122.8	122.5	122.8	122.0
R(2-13)	1.347	1.347	1.755	1.356	A(5-40-7)	118.6	118.6	118.7	116.2
R(3-34)	1.355	1.354	1.766	1.370	A(5-40-39)	124.8	124.8	124.8	126.4
R(4-23)	1.224	1.224	1.223	1.216	A(6-42-8)	116.7	116.7	116.8	117.8
R(5-40)	1.237	1.236	1.237	1.254	A(6-42-39)	124.8	124.6	124.7	124.3
R(6-42)	1.286	1.289	1.285	1.256	A(40-7-41)	124.5	124.5	124.5	124.1
R(7-40)	1.433	1.432	1.432	1.421	A(40-7-43)	115.0	115.0	115.0	116.1
R(7-41)	1.373	1.373	1.373	1.366	A(7-40-39)	116.6	116.6	116.5	117.4
R(7-43)	1.479	1.480	1.480	1.474	A(41-7-43)	120.5	120.5	120.5	119.6
R(8-41)	1.384	1.385	1.385	1.369	A(7-41-8)	116.1	116.1	116.2	116.1
R(8-42)	1.425	1.424	1.425	1.415	A(7-43-46)	112.2	112.2	112.3	111.2
R(8-50)	1.478	1.478	1.478	1.475	A(41-8-42)	123.5	123.4	123.5	123.6
R(9-11)	1.389	1.389	1.389	1.388	A(41-8-50)	119.5	119.5	119.5	119.2
R(9-18)	1.410	1.410	1.409	1.403	A(42-8-50)	116.8	116.9	116.8	117.0
R(11-13)	1.393	1.393	1.397	1.368	A(8-42-39)	118.5	118.7	118.4	117.9
R(13-14)	1.390	1.390	1.394	1.368	A(8-50-53)	114.1	114.1	114.0	112.2
R(14-16)	1.392	1.392	1.392	1.387	A(11-9-18)	121.2	121.2	121.3	121.3
R(16-18)	1.408	1.408	1.407	1.394	A(9-11-13)	118.8	118.8	119.3	117.7
R(18-19)	1.462	1.461	1.462	1.462	A(9-18-16)	117.9	118.0	117.8	118.4
R(19-21)	1.348	1.347	1.347	1.334	A(9-18-19)	123.3	123.3	123.4	122.7
R(21-23)	1.491	1.488	1.492	1.472	A(11-13-14)	121.9	122.0	121.0	123.3
R(23-24)	1.521	1.523	1.521	1.513	A(13-14-16)	118.5	118.5	119.0	118.7
R(24-27)	1.537	1.537	1.537	1.525	A(14-16-18)	121.6	121.6	121.6	120.6
R(27-29)	1.531	1.531	1.529	1.527	A(16-18-19)	118.8	118.7	118.8	118.9
R(27-39)	1.525	1.524	1.524	1.513	A(18-19-21)	128.4	128.3	128.2	126.0
R(29-30)	1.399	1.400	1.399	1.389	A(19-21-23)	120.7	120.7	120.7	122.7
R(29-37)	1.407	1.407	1.406	1.393	A(21-23-24)	115.5	115.6	115.5	115.0
R(30-32)	1.400	1.401	1.399	1.396	A(23-24-27)	115.5	115.2	115.5	114.8
R(32-34)	1.387	1.388	1.391	1.368	A(24-27-29)	114.9	115.0	114.9	113.9
R(34-35)	1.392	1.392	1.396	1.368	A(24-27-39)	111.0	111.2	111.0	112.0
R(35-37)	1.394	1.394	1.394	1.385	A(29-27-39)	113.6	113.7	113.4	111.1
R(39-40)	1.430	1.431	1.430	1.391	A(27-29-30)	124.0	124.2	124.1	124.2
R(39-42)	1.387	1.386	1.387	1.397	A(27-29-37)	118.3	118.1	118.3	117.9
R(43-46)	1.528	1.528	1.528	1.514	A(27-39-40)	117.1	117.2	117.2	119.5
R(50-53)	1.528	1.529	1.528	1.513	A(27-39-42)	122.5	122.5	122.4	119.9
R(57-62)	1.501	1.501	1.501	1.487	A(30-29-37)	117.7	117.6	117.6	117.9
R(57-65)	1.501	1.498	1.501	1.488	A(29-30-32)	121.7	121.6	121.7	121.1
R(58-62)	1.523	1.523	1.523	1.507	A(29-37-35)	121.6	121.7	121.7	121.7
R(65-68)	1.523	1.524	1.523	1.508	A(30-32-34)	118.7	118.8	119.1	118.2
A(1-41-7)	122.3	122.3	122.3	122.3	A(32-34-35)	121.5	121.5	120.7	122.9
A(1-41-8)	121.5	121.5	121.5	121.6	A(34-35-37)	118.8	118.8	119.2	118.1
A(2-13-11)	118.9	118.9	119.4	118.6	A(40-39-42)	120.4	120.3	120.5	120.6
A(2-13-14)	119.2	119.2	119.6	118.1	A(62-57-65)	114.3	114.4	114.3	113.9
A(3-34-32)	119.3	119.3	119.6	118.5	A(57-62-58)	111.9	111.8	112.0	110.4
A(3-34-35)	119.2	119.3	119.7	118.6	A(57-65-68)	111.3	110.9	111.3	110.5
A(4-23-21)	121.7	122.0	121.7	123.0					

**^a^** For atom numbering, refer to Figure 3.

The distribution of atomic charges is strongly related to the electronic properties of compound [24]. The calculated natural charges (NAC) are given in Table 5. The O, S and N-atoms have electronegative nature. The highest NAC occurs at the O-atoms which are responsible for the high polarity of the studied compounds. The dipole moment values of **3a**, **3b** and **3c** were calculated to be 13.62D, 11.92D and 13.04D, respectively. Eliminating the methanol molecule from the structure of **3a** decreases the dipole moment while exchanging the halogen substituent showed almost no effect on the polarity of the studied systems. On other hand, the compound **3c** showed a lower electronegative nature for the Cl-atom compared to fluorine. On other hand, the negative natural charge values at the O4, O5 and O6 atoms are decreased in **3c** compared to the others. In contrast, the N7 and N8 atoms are more negative in **3c**.

**Table 5:** The B3LYP/6-31G(d) calculated natural charges.

Atom	3a	3b	3c	Atom	3a	3b	3c
S1	−0.2768	−0.2719	−0.3166	C41	0.2791	0.2778	0.3354
F (Cl)2	−0.3284	−0.3287	(−0.0121)	C42	0.6153	0.6144	0.5547
F (Cl)3	−0.3414	−0.3389	(−0.0473)	C43	−0.2630	−0.2632	−0.1403
O4	−0.5583	−0.5598	−0.4727	H44	0.2599	0.2605	0.1816
O5	−0.6582	−0.6546	−0.5683	H45	0.2577	0.2580	0.1864
O6	−0.7931	−0.7953	−0.6889	C46	−0.6861	−0.6863	−0.4477
N7	−0.4497	−0.4495	−0.5040	H47	0.2332	0.2341	0.1434
N8	−0.4511	−0.4511	−0.4954	H48	0.2361	0.2361	0.1645
C9	−0.1856	−0.1845	−0.1721	H49	0.2332	0.2332	0.1564
H10	0.2391	0.2384	0.1436	C50	−0.2637	−0.2643	−0.1389
C11	−0.2998	−0.2998	−0.1306	H51	0.2621	0.2628	0.1924
H12	0.2538	0.2533	0.1601	H52	0.2514	0.2512	0.1659
C13	0.4323	0.4327	−0.0649	C53	−0.6750	−0.6752	−0.4451
C14	−0.3021	−0.3019	−0.1318	H54	0.2332	0.2342	0.1444
H15	0.2549	0.2548	0.1619	H55	0.1875	0.1858	0.0970
C16	−0.1854	−0.1842	−0.1868	H56	0.2550	0.2562	0.1955
H17	0.2443	0.2443	0.1519	N57	−0.6390	−0.6411	−0.6438
C18	−0.1037	−0.1032	0.1717	C58	−0.7043	−0.7042	−0.4778
C19	−0.1471	−0.1425	−0.1564	H59	0.2594	0.2603	0.1823
H20	0.2503	0.2498	0.1704	H60	0.2575	0.2573	0.2000
C21	−0.3233	−0.3204	−0.2074	H61	0.2321	0.2329	0.1597
H22	0.2216	0.2203	0.1306	C62	−0.2595	−0.2599	−0.1682
C23	0.5456	0.5440	0.4455	H63	0.2334	0.2345	0.1685
C24	−0.5416	−0.5400	−0.4057	H64	0.2671	0.2661	0.2257
H25	0.2705	0.2700	0.1740	C65	−0.2604	−0.2585	−0.1658
H26	0.2575	0.2553	0.1759	H66	0.2725	0.2578	0.2305
C27	−0.2774	−0.2793	−0.2173	H67	0.2309	0.2365	0.1638
H28	0.3017	0.3043	0.1997	C68	−0.7069	−0.7080	−0.4834
C29	−0.0184	−0.0165	0.1929	H69	0.2591	0.2544	0.1814
C30	−0.2356	−0.2376	−0.2040	H70	0.2269	0.2318	0.1490
H31	0.2364	0.2361	0.1371	H71	0.2615	0.2653	0.2055
C32	−0.3189	−0.3196	−0.1538	H72	0.4312	0.4317	0.3686
H33	0.2480	0.2473	0.1414	H73	0.4901	0.4902	0.4600
C34	0.3958	0.3977	−0.0780	C74	−0.3071		−0.2206
C35	−0.3077	−0.3082	−0.1371	H75	0.1922		0.1420
H36	0.2497	0.2511	0.1531	H76	0.1981		0.1510
C37	−0.2096	−0.2103	−0.1905	H77	0.2179		0.1726
H38	0.2606	0.2606	0.1761	O78	−0.7670		−0.6131
C39	−0.2761	−0.2740	−0.0583	H79	0.4766		0.3999
C40	0.6489	0.6496	0.5807				

Interestingly, the calculated natural charge value at C39 showed that this atom has a partial negative charge of −0.2761 unit inspite the presence of this C-atom between two carbonyl groups. The small negative natural charge value at this carboanion confirms the presence of electron delocalization with the adjacent C=O π-system. In this regard, natural bond orbital (NBO) calculation was performed. The second order perturbation energies E^(2)^ obtained from the NBO calculations offers quantitative description for the intramolecular charge transfer (ICT) interactions [25,26]. The E^(2)^ value represents the stabilization energy due to this electronic delocalization. The NBO analysis showed strong electron delocalization (E^(2)^ = 18.4 KJ/mol) from the π-NBO of the C40–O5 to the adjacent empty π*-NBO of the C39=C42 bond. The presence of such electron delocalization could be the reason for the stabilization of **3**.

### 3.3. Frontier Molecular Orbitals (FMOs)

**Figure 4:**
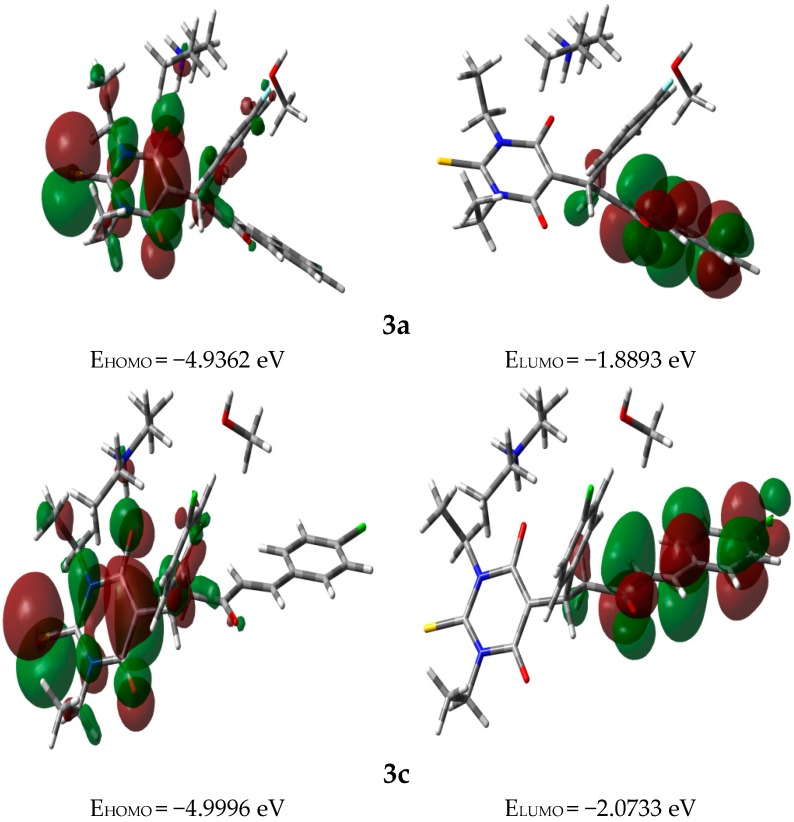
Ground state isodensity surface plots for the frontier molecular orbitals.

For chemists and physicists, the properties of these molecular orbitals (FMOs) like energy and electron densities are very important quantum chemical parameters. They were used to predict the most reactive position in conjugated π-system [27]. The HOMO and LUMO levels are located on the π-system of **3** (Figure 4). Moreover, the energies of the FMOs and their energy gap (ΔE) are related to the chemical reactivity and bioactivity of the compound [28,29]. A molecule having high ΔE value has low chemical reactivity [28]. The energies of these FMOs for **3** were calculated using the same method. The E_HOMO_ and E_LUMO_ values of compounds **3a** and **3c** are shown in Figure 4. The F-substituted molecule (**3a**) has higher energy gap (3.0469 eV) than the Cl-substituted one (2.9263 eV). The former (**3a**) has higher stability towards electron transfer process more than the latter, hence **3c** is more polarizable than **3a**.

Based on the energies of the FMOs, various chemical reactivity descriptors such as electronegativity (χ), chemical potential (μ), chemical hardness (η), global softness (S) and global electrophilicity index (ω) [30,31,32,33,34] were proposed for understanding the different pharmacological aspects of drug molecules. These descriptors are calculated using Equations (1)–(5) given below:

(1) $$\text{χ}=\frac{\left(\text{I}+\text{A}\right)}{2}$$

(2) $$\text{μ}=-\text{χ}=-\frac{\left(\text{I}+\text{A}\right)}{2}$$

(3) $$\text{η}=\frac{\left(\text{I}-\text{A}\right)}{2}$$

(4) $$\text{S}=\frac{1}{2\;\eta }$$

(5) $$\text{ω}=\;\frac{{\mu \;}^{2}}{2\;\eta }$$

The hardness (S) indicates the resistance to charge transference [35] while the electrophilicity index (ω) measures the stabilization in energy when the system acquires an additional charge. For the studied compounds, these reactivity descriptors are shown in Table 6. It is seen that all the studied systems have negative chemical potentials means that the compounds are stable. In addition, the fluorinated compound (**3a**) is harder than the chloro one. Moreover, compound **3c** has lower μ and higher ω than **3a** indicating the higher electrophilic behavior of **3a** [36]. These results are in accord with the higher electronegativity of the F-atom compared to chlorine.

**Table 6:** Calculated reactivity descriptors (in eV) for the studied compound.

Compound	χ	μ	Η	S	ω
3a	−3.4127	3.4127	1.5234	0.3282	3.8225
3c	−3.5364	3.5364	1.4632	0.3417	4.2737

### 3.4. Electronic Spectra and TD-DFT Calculations

**Figure 5:**
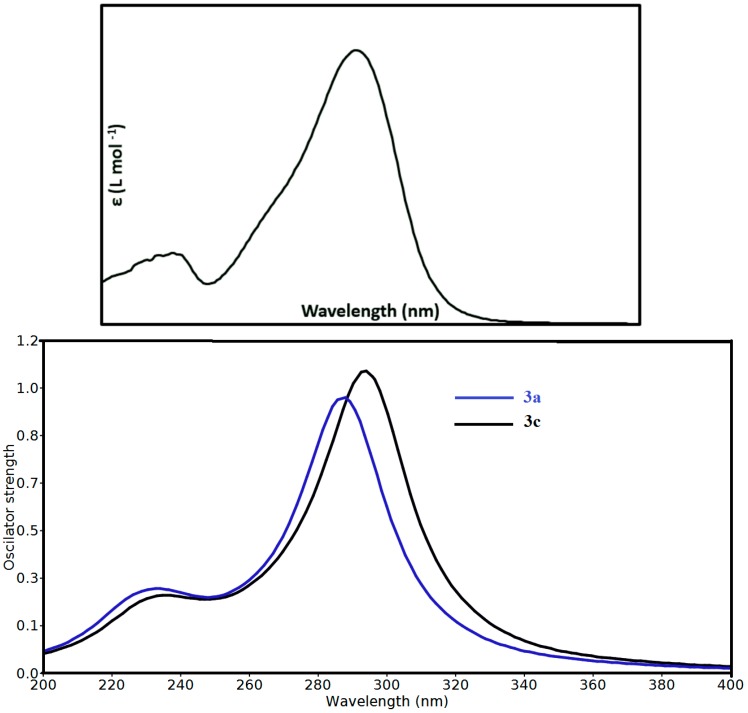
Experimental (**upper**) and calculated (**lower**) electronic spectra of the studied compounds using TD-DFT method.

The electronic spectrum of **3a** (1.7370 ×10^−5^ M, dichloromethane) is shown in Figure 5. Two electronic transition bands of variable intensities showed good agreement with our calculated electronic spectra. Figure 5 showed an intense and broad electronic transition band at 294.0 nm with high molar absorptivity (ε = 21326 L·Mol^−1^). In addition, a small band was observed at 228.0 nm (ε = 5591 L·Mol^−1^). The TD‒DFT method was used to calculate the accurate electronic transitions of **3** (Table S1; Supplementary Information). The computationally derived spectra of compounds **3a** and **3c** are shown in Figure 5. In agreement with the experimental electronic spectra, the former showed a strong band at 287.9 nm (A = 0.827) due to the H-5/H-4→L and H→L+3 transitions. Moreover, a transition band at higher energy and lower absorption intensity has been predicted at 236.5 nm (A = 0.082) due to H-2→L+3 excitation. Compound **3c** showed these two transition bands at 292.7 nm (A = 0.676) and 233.1 nm A = 0.051), respectively. The most intense band occurred at longer wavelength in case of **3c**.

### 3.5. Charge Decomposition Analysis (CDA)

Charge decomposition analysis [37] was used to provide insight into the mechanism of charge transfer between fragments in this molecular system. In this approach, the fragment orbital (FO) represents the molecular orbital (MO) of the molecular fragment in its isolated state. The studied compounds, **3a** and **3c**, have three fragments, methanol, the diethyl ammonium cation [EtNH_2_]^+^ and the organic anionic part [OAP]^−^. The CDA of the three fragments of the studied compound showed that the [OAP]^−^ highest occupied FO contributes 79.5%to the HOMO composition of **3** while the [OAP]^−^ lowest unoccupied FO contributes 98.0% to composition of the LUMO. Hence, the orbital interactions between the fragments are considered to be weak, since neither the methanol nor [EtNH_2_]^+^ fragments contribute very much to the frontier molecular orbitals of **3**.

In CDA, the terms d_i_, b_i_ and r are important and must be examined. The term d_i_ is defined as electron donation between two fragments (A→B) via MO i of the complex; the term b_i_ is the electron back-donation (B→A). Hence, the difference between d and b is that which fragment provides its electrons from its occupied FOs to virtual FOs of another fragment. The term r refers to closed–shell interactions between two occupied FOs in different fragments. The most significant CDA results were collected in Table S2 (Supplementary material). The analyses of **3** showed that the charge transfer between [EtNH_2_]^+^ and [OAP]^−^ is the most important. The charge transfers between the methanol fragment and either [EtNH_2_]^+^ or [OAP]^−^ are very small. The results indicate that the total amount of electron donation from the fragment [OAP]^−^ to [EtNH_2_]^+^ is 0.1717 e^−^ and 0.1673 e^−^ for **3a** and **3c**, respectively. On other hand, the amount of electron back-donation from [EtNH_2_]^+^ to [OAP]^−^ is only 0.0121 e^−^ and 0.0122 e^−^ for **3a** and **3c**, respectively. The net charge transfer between the two fragments is almost equal, 0.1596 e^−^; **3a** and 0.1551 e^−^; **3c**, where the direction of electron donation occurs from [OAP]^−^ as donor to [EtNH_2_]^+^ as acceptor.

### 3.6. Molecular Docking Study

**Figure 6:**
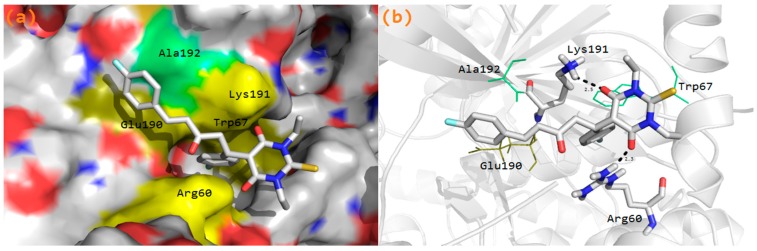
(**a**) Compound **3a** fits to the active site cavity of GPb enzyme; (**b**) Binding conformation of compound **3a** in the active site of GPb enzyme.

PASS (Prediction of Activity Spectra) is an online tool [38] which predicts nearly 900 different types of activities on the basis of molecule structure. Prediction of Activity Spectra (Supplementary Table S3) of **3** predicts anti-diabetic activity with a Pa (probability to be active) value = 0.229. Glycogen phosphorylase b has been identified as a target for anti-diabetic drug [39]. Molecular docking simulations were performed to examine the inhibitory nature of the molecule against glycogen phosphorylase b (GPb). Molecular docking is a method for recognizing ligand-receptor interactions at a glance. Molecular docking studies were carried out using the Molecular Operating Environment (MOE) docking software [40]. The three dimensional (3D) crystal structure of GPb was downloaded from the Protein Data Bank (PDB ID: 1C50) [41]. The allosteric site as characterized in the crystal structure of GPb was used as the target site. Before the docking experiment was performed, the compounds prepared for docking by minimizing its energy at the B3LYP/631G(d) level of theory. Partial charges were calculated by Gasteiger’s method. Since most macromolecular crystal structures contain little or no hydrogen coordinate data due to limited resolution, protonation was done prior to docking using Protonate 3D tools implemented in MOE. Protonation was followed by energy minimization up to a 0.05 Gradient using an Amber 99 force field. The compounds were docked into the protein’s active site using the Triangular Matching docking method and thirty conformations of the title compound and protein complex were generated with a docking score (S). The complexes were analyzed for interactions and their 3D images were taken by using visualizing tool PyMol. The docking protocol predicted the same conformation as was present in the crystal structure with an RMSD value within the allowed range of 2.0 Å [42]. The superposition of the docked conformation and co-crystalized ligand is shown in Figure S1 (supplementary materials). Amongst the docked conformations, the top-ranked conformation was visualized for ligand-enzyme interactions in PyMol software. Analysis of the docking results showed that the synthesized compounds fit well within the active site of GPb (Figure 6a). From the docking study, it was observed that both the carbonyl oxygen groups of pyrimidine moiety and phenyl ring of the compound **3a** interacts with important active site residues of the enzyme, e.g., Arg60 and Lys191. Arg60 was found to interact with both the phenyl ring (arene-cation) and carbonyl oxygen (H-Bond side chain donor) of the compound, while Lys191 interacted with the carbonyl oxygen of pyrimidine moiety (Figure 6b) of compound **3a**.

**Figure 7:**
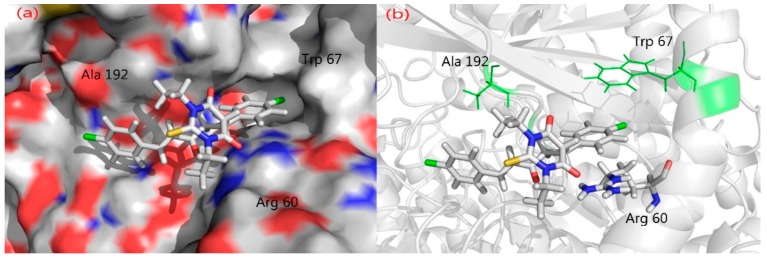
(**a**) Compound **3c** fits to the active site cavity of GPb enzyme; (**b**) Binding conformation of compound **3c** in the active site of GPb enzyme.

In case of chloro substituted compound, only arene-arene interaction was observed between Arg60 and chlorophenyl ring of the compound (Figure 7b). The docking results showed that fluorine containing compound might be more active as compared to chlorine containing compound as shown in Figure 7a,b. These preliminary observations suggest that the compound may exhibit inhibitory activity against GPb and thus might act as potent anti-diabetic compound. However, further biological tests should be done to validate the computational predictions. Hence, the fluorine atom incorporation into the molecular structure results in greater biological activity than the other halogenated analogue, leading to an improvement in the biological availability.

## 4. Materials and Methods

### 4.1. General

Chemicals were purchased from Fluka and Aldrich Chemical companies, and were used without further purification, unless otherwise stated. The progress of the reaction was monitored by TLC. IR Spectra were measured as KBr pellets on a Nicolet 6700 FT-IR spectrophotometer. The NMR spectra were recorded on a Jeol-400 NMR spectrometer. ^1^H-NMR (400 MHz), and ^13^C-NMR (100 MHz) were run in *d*_6_-DMSO.Chemical shifts (δ) are reported in terms *ppm* relative to TMS and *J*-coupling constants are given in *Hz*. The X-ray crystallographic analysis of compound **3** was collected by using Bruker SMART APEXII D8 Venture diffractometer. Mass spectrometric analysis was conducted by using ESI mode on AGILENT Technologies 6410–triple quad LC/MS instrument.

### 4.2. Synthesis of Diethylammonium (E)-5-(1,5-bis(4-Fluorophenyl)-3-oxopent-4-en-1-yl)-1,3-diethyl-4,6-dioxo-2-thioxohexahydropyrimidin-5-ide ***3***

To a 50 mL round bottom flask is added DCM (10 mL), *N*,*N*-diethyl thiobarbituric acid **1** (400 mg, 2 mmol) and (1*E*,4*E*)-1,5-*bis*(4-fluorophenyl)penta-1,4-dien-3-one **2** (540 mg, 2 mmol) and placed under an N_2_ atmosphere with stirring. The Et_2_NH (258 μL, 2.5 mmol) was then added dropwise and the reaction mixture further stirred for 30 min. Upon completion of the reaction (as determined by TLC), the product was precipitated by addition of 25 mL of petroleum ether. The solid product was crystalized from a 1:1:1 mixture of DCM/EtOH/Et_2_O to afford 675 mg of the pure product, **3**, as white crystals, m.p. 120–122 °C in 90% yield.

^1^H-NMR (400 MHz, *d*_6_-DMSO) δ: 0.77 (t, 3H, *J* = 6.6 Hz, OCNCH_2_CH_3_), 1.05 (t, 3H, *J* = 6.6 Hz, OCNCH_2_CH_3_), 1.15 (t, 6H, *J* = 7.36 Hz, NCH_2_CH_3_), 2.90 (q, 2H, *J* = 7.3 Hz, NHCH_2_CH_3_), 3.42 (dd, 1H, *J* = 7.3 Hz, 1.5 Hz, CH_2_), 3.59 (dd, 1H, *J* = 7.3 Hz, 1.5 Hz, CH_2_), 3.90 (t, 1H, *J* = 7.3 Hz, CHCH_2_CO), 4.11 (q, 2H, *J* = 7.3 Hz, OCNCH_2_CH_3_), 4.32 (q, 2H, *J* = 7.3 Hz, OCNCH_2_CH_3_), 6.86 (d, 1H, *J* = 7.3 Hz, CH=CH), 6.93 (t, 1H, *J* = 7.3 Hz, Ar-H), 7.10 (d, 4H, *J* = 6.6 Hz, Ar-H), 7.23 (d, 1H, *J* = 7.36 Hz, Ar-H), 7.39 (dd, 1H, *J* = 7.3, 2.9 Hz, Ar-H), 7.41 (d, 1H, *J* = 7.3 Hz, CH=CH), 7.71 (dd, 1H, *J* = 7.3, 2.9 Hz, Ar-H), 8.23 (bs, 2H, NH_2_CH_2_CH_3_)_2_); ^13^C-NMR (100 MHz, *d*_6_-DMSO) δ: 206.4,199.8, 177.1, 173.9, 168.4, 166.4, 160.5, 160.2, 143.0, 139.6, 129.8, 129.8, 129.2, 129.1, 115.7, 115.4, 113.7,92.9, 60.3, 49.9, 43.6, 43.2, 43.1,12.8, 11.0; IR (KBr, cm^−1^) ν_max_ = 3410, 2979, 1666, 1573,1508, 1398, 1265; Anal. Calcd. for C_29_H_35_F_2_N_3_O_3_S: C, 64.07; H, 6.49; F, 6.99; N, 7.73; S, 5.90;Found: C, 64.09; H, 6.48; F, 7.01; N, 7.75; S, 5.91; LC/MS (ESI, *m*/*z*): [M^+^], found 543.35, C_29_H_35_F_2_N_3_O_3_S requires 543.24; Uv-vis (DCM): 294.0 nm.

### 4.3. Single-Crystal X-Ray Diffraction Studies

Compound **3** was obtained as single crystals by slow evaporation of diethyl ether solution of the pure compound **3** in dichloromethane at room temperature for 24 h. Data were collected on a Bruker APEX-II D8 Venture area diffractometer (Bruker AXS GmbH, Karlsruhe, Germany), equipped with graphite monochromatic Mo Kα radiation at 150(2) °K. Cell refinement and data reduction were carried out by Bruker SAINT. SHELXS-97 [43,44,45] was used to solve structure. The final refinement was carried out by full-matrix least-squares techniques with anisotropic thermal data for nonhydrogen atoms on *F*^2^.

### 4.4. Computational Methods

Geometries for the studied compounds were optimized using the B3LYP method and 6–31G(d) basis set. The calculations were started using the atomic coordinates obtained from the X-ray crystallographic file (CIF) as input files. The calculations were made using Gaussian 03 software [46] with the help of GaussView4.1 [47] and Chemcraft [48] programs. No symmetry restrictions were applied and the geometries were optimized followed by frequency calculations at the optimized geometries. No imaginary frequencies were obtained, indicating a real energy minimum at the optimized structure. The electronic spectra of the studied compounds were calculated by the TD–DFT method. The natural atomic charges were calculated using Gaussian NBO Version 3.1 [49].

## 5. Conclusions

In conclusion, a regioselective synthesis of adduct **3** via Michael addition reaction using diethylamine as a base at room temperature is reported. An initio structural studies using B3LYP/6-31G(d) method were conducted on **3** with methanol solvent, without methanol solvents how that calculated bond distances and bond angles of **3** agree well with the experimental structure. In addition, computations for the chloro-substituted derivative were performed that show only a modest shift in structural parameters arising from chlorine substitution for fluorine. The charge densities were calculated using NBO method and show that when chlorine is substituted for fluorine, the molecular dipole moment is reduced by ~4%. The electronic transitions associated with the π-π* excitation from the pyrimidine moiety to the benzoyl ring were calculated using TD-DFT method and are consistent with molecules having extended conjugation. Further bioactivity studies of compound **3** are underway and will be reported in a separate communication.

## Supplementary Materials

Supplementary materials can be accessed at: http://www.mdpi.com/1420-3049/20/11/19710/s1.
