# Supplementary material for: New Diethyl Ammonium Salt of Thiobarbituric Acid Derivative: Synthesis, Molecular Structure Investigations and Docking Studies
Source: Molecules. 2015 Nov 19;20(11):20642–58. doi: 10.3390/molecules201119710 (PMC6331823; doi:10.3390/molecules201119710)
Supplement: Supplementary file 1 [file molecules-20-19710-s001.pdf]

Supplementary Materials: New Diethyl Ammonium Salt of Thiobarbituric Acid Derivative: Synthesis, Molecular Structure Investigations and Docking Studies

Assem Barakat, Abdullah Mohammed Al-Majid, Saied M. Soliman, Gehad Lotfy, Hazem A. Ghabbour, Hoong-Kun Fun, Abdul Wadood, Ismail Warad and Joseph C. Sloop

Table S1. The calculated electronic transitions using TD-DFT method.

λ_{max}(nm)	f	Major Contributions
		**3a**
473.4	0.003	H→L (97%)
434.6	0.000	H-1→L (98%)
374.0	0.002	H-6→L (46%), H-5→L (12%), H-3→L (36%)
352.8	0.002	H-2→L (99%)
327.4	0.001	H-1→L+2 (21%), H-1→L+3 (64%)
319.1	0.010	H-6→L (32%), H-3→L (60%)
307.2	0.000	H→L+1 (99%)
304.0	0.008	H-6→L (15%), H-5→L (42%), H-4→L (42%)
297.1	0.059	H→L+2 (90%)
295.2	0.000	H-1→L+1 (99%)
287.9	0.827	H-5→L (35%), H-4→L (42%), H→L+3 (16%)
281.4	0.134	H→L+3 (62%)
276.9	0.001	H-7→L (91%)
275.4	0.001	H-1→L+2 (70%), H-1→L+3 (24%)
273.9	0.005	H-8→L (90%)
271.6	0.017	H→L+4 (83%)
266.9	0.003	H-1→L+4 (30%), H-1→L+6 (26%), H-1→L+7 (12%), H→L+6 (14%)
264.4	0.006	H-10→L (60%), H-5→L+1 (12%), H-4→L+1 (21%)
262.2	0.033	H-1→L+6 (10%), H→L+6 (60%)
257.2	0.003	H-9→L (98%)
254.3	0.005	H→L+5 (51%), H→L+6 (17%), H→L+7 (23%)
253.5	0.002	H-2→L+1 (79%), H→L+7 (13%)
253.3	0.001	H-2→L+1 (11%), H-1→L+4 (48%), H-1→L+6 (17%)
252.8	0.007	H→L+5 (29%), H→L+7 (45%)
245.1	0.002	H-1→L+6 (29%), H-1→L+7 (65%)
243.2	0.015	H-2→L+2 (88%)
242.6	0.000	H-1→L+5 (90%)
240.1	0.002	H-3→L+1 (84%)
236.5	0.082	H-2→L+3 (84%)
236.0	0.012	H-8→L+4 (10%), H-3→L+2 (72%)
232.7	0.012	H-5→L+3 (12%), H-4→L+3 (17%), H-3→L+3 (24%)
230.5	0.000	H→L+8 (96%)
229.4	0.013	H-2→L+4 (10%)
228.7	0.003	H-6→L+1 (29%), H-5→L+1 (38%), H-4→L+1 (18%)
226.3	0.082	H-10→L (28%), H-4→L+1 (55%)
224.8	0.015	H-2→L+4 (80%)
224.1	0.023	H-6→L+1 (47%), H-5→L+1 (37%)
222.6	0.007	H-5→L+2 (22%), H-4→L+2 (18%), H-3→L+3 (45%)
221.8	0.000	H-1→L+8 (95%)
220.9	0.008	H-6→L+2 (24%), H-5→L+3 (14%)

Table S1. *Cont.*

λ_{max}(nm)	f	Major Contributions
		**3c**
494.5	0.003	H→L (96%)
455.2	0.000	H-1→L (98%)
379.2	0.002	H-6→L (46%), H-3→L (34%)
365.1	0.002	H-2→L (98%)
328.5	0.001	H-1→L+2 (10%), H-1→L+3 (34%), H-1→L+4 (42%)
324.1	0.011	H-6→L (25%), H-4→L (11%), H-3→L (62%)
311.5	0.015	H-6→L (21%), H-4→L (69%)
307.0	0.000	H→L+1 (98%)
300.2	0.003	H→L+2 (81%), H→L+3 (13%)
296.5	0.391	H-5→L (15%), H→L+3 (63%)
295.9	0.000	H-1→L+1 (99%)
292.7	0.676	H-5→L (63%), H-4→L (12%), H→L+3 (12%)
284.3	0.000	H-7→L (98%)
279.5	0.000	H-1→L+2 (77%), H-1→L+3 (17%)
278.6	0.005	H-8→L (97%)
276.5	0.083	H→L+4 (78%)
272.4	0.001	H-1→L+2 (10%), H-1→L+3 (35%), H-1→L+4 (46%)
268.3	0.004	H-10→L (66%), H-5→L+1 (23%)
265.9	0.011	H-1→L+6 (15%), H→L+5 (25%), H→L+6 (37%)
264.8	0.001	H-1→L+6 (21%), H→L+5 (57%)
263.6	0.006	H-9→L (89%)
262.2	0.029	H-1→L+6 (26%), H→L+6 (42%)
253.6	0.000	H-2→L+1 (97%)
253.4	0.000	H-1→L+5 (84%)
253.1	0.014	H→L+7 (79%)
245.6	0.004	H-1→L+6 (21%), H-1→L+7 (64%)
244.9	0.008	H-2→L+2 (81%)
241.6	0.024	H-2→L+3 (87%)
238.2	0.007	H-3→L+1 (23%), H-3→L+2 (37%)
237.5	0.009	H-3→L+1 (52%), H→L+9 (24%)
237.2	0.005	H-3→L+2 (13%), H→L+9 (63%)
234.8	0.028	H-2→L+4 (56%)
234.0	0.000	H→L+8 (99%)
233.1	0.051	H-4→L+3 (18%), H-3→L+3 (15%), H-2→L+4 (25%)
228.7	0.019	H-4→L+2 (13%), H-4→L+6 (15%), H-3→L+4 (10%)
228.2	0.000	H-1→L+8 (93%)
228.1	0.004	H-6→L+1 (14%), H-5→L+1 (14%), H-4→L+1 (39%), H-3→L+1 (10%)
227.9	0.003	H-1→L+9 (80%)
226.9	0.066	H-10→L (25%), H-5→L+1 (30%), H-4→L+1 (28%)
225.2	0.003	H-6→L+2 (15%), H-6→L+5 (15%), H-4→L+1 (11%), H-3→L+5 (20%)

Table S2. The b, d and r values of the charge decomposition analysis of the fragments $[EtNH_2]^+$ to $[OAP]^-$ of **3.**

	d	b	d-b	r
		**3a**		
HOMO	0.0053	0.0000	0.0053	−0.0047
HOMO-2	0.0058	0.0000	0.0058	−0.0063
HOMO-3	0.0033	−0.0001	0.0034	−0.0025
HOMO-7	0.0114	−0.0002	0.0116	−0.0184
HOMO-8	0.0103	0.0000	0.0102	−0.0114
		**3c**		
HOMO	0.0020	0.0001	0.0021	−0.0058
HOMO-1	0.0011	0.0000	0.0011	−0.0019
HOMO-2	0.0066	0.0013	0.0053	−0.0051
HOMO-5	0.0043	0.0015	0.0028	−0.0177
HOMO-6	0.0020	0.0001	0.0021	−0.0058
HOMO-7	0.0011	0.0000	0.0011	−0.0019

Table S3. PASS prediction of the compound, Pa represents probability to be active and Pi represents probability to be inactive.

Pa	Pi	Predicted activity
0.279	0.139	Menopausal disorders treatment
0.232	0.093	UGT2B4 substrate
0.286	0.149	Neurodegenerative diseases treatment
0.211	0.074	Tankyrase inhibitor
0.157	0.022	Histone deacetylase SIRT2 inhibitor
0.229	0.094	Antidiabetic symptomatic
0.218	0.085	Morphine 6-dehydrogenase inhibitor
0.145	0.012	Acetylcholine release stimulant
0.285	0.154	CYP2A1 substrate
0.328	0.201	CYP3A2 substrate
0.253	0.127	CYP2B5 substrate
0.179	0.053	Contraceptive female
0.299	0.176	Thioredoxin inhibitor
0.155	0.033	Phosphorylase b inhibitor
0.156	0.035	Narcolepsy treatment

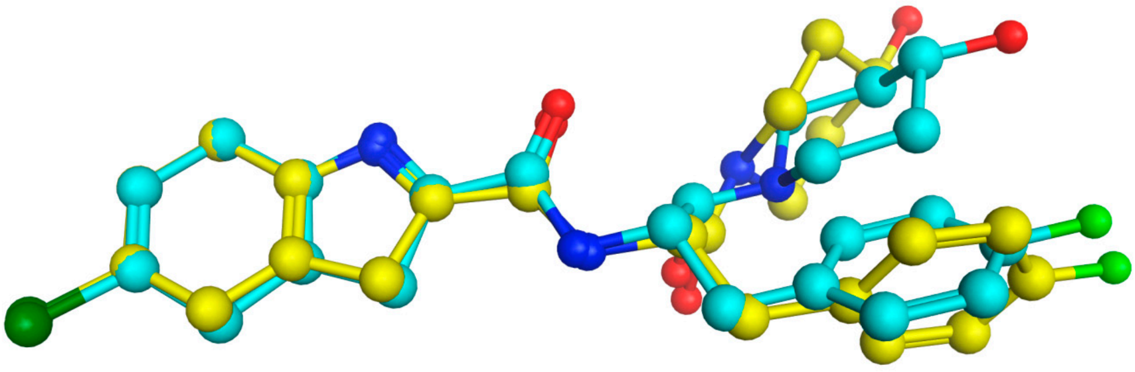

Figure S1. The superposition of the docked conformation and co-crystalized ligand.
